# A complex endeavour: an ethnographic study of the implementation of the Sepsis Six clinical care bundle

**DOI:** 10.1186/s13012-016-0518-z

**Published:** 2016-11-16

**Authors:** Carolyn Tarrant, Barbara O’Donnell, Graham Martin, Julian Bion, Alison Hunter, Kevin D. Rooney

**Affiliations:** 1 SAPPHIRE, Department of Health Sciences, University of Leicester, Leicester, UK; 2 Institute of Healthcare Policy and Practice, School of Health, Nursing and Midwifery, University of the West of Scotland, Paisley, UK; 3 University Department of Anaesthesia and Critical Care, University of Birmingham, Birmingham, UK; 4 Healthcare Improvement Scotland, Glasgow, UK; 5 Department of Anaesthesia and Intensive Care Medicine, Royal Alexandra Hospital, Paisley, UK

## Abstract

**Background:**

Implementation of the ‘Sepsis Six’ clinical care bundle within an hour of recognition of sepsis is recommended as an approach to reduce mortality in patients with sepsis, but achieving reliable delivery of the bundle has proved challenging. There remains little understanding of the barriers to reliable implementation of bundle components. We examined frontline clinical practice in implementing the Sepsis Six.

**Methods:**

We conducted an ethnographic study in six hospitals participating in the Scottish Patient Safety Programme Sepsis collaborative. We conducted around 300 h of non-participant observation in emergency departments, acute medical receiving units and medical and surgical wards. We interviewed a purposive sample of 43 members of hospital staff. Data were analysed using a constant comparative approach.

**Results:**

Implementation strategies to promote reliable use of the Sepsis Six primarily focused on education, engaging and motivating staff, and providing prompts for behaviour, along with efforts to ensure that equipment required was readily available. Although these strategies were successful in raising staff awareness of sepsis and engagement with implementation, our study identified that completing the bundle within an hour was not straightforward. Our emergent theory suggested that rather than being an apparently simple sequence of six steps, the Sepsis Six actually involved a complex trajectory comprising multiple interdependent tasks that required prioritisation and scheduling, and which was prone to problems of coordination and operational failures. Interventions that involved allocating specific roles and responsibilities for completing the Sepsis Six in ways that reduced the need for coordination and task switching, and the use of process mapping to identify system failures along the trajectory, could help mitigate against some of these problems.

**Conclusions:**

Implementation efforts that focus on individual behaviour change to improve uptake of the Sepsis Six should be supplemented by an understanding of the bundle as a complex trajectory of work in which improving reliability requires attention to coordination of workflow, as well as addressing the mundane problems of interruptions and operational failures that obstruct task completion.

**Electronic supplementary material:**

The online version of this article (doi:10.1186/s13012-016-0518-z) contains supplementary material, which is available to authorized users.

## Background

Sepsis is a “life-threatening organ dysfunction caused by a dysregulated host response to infection” [1]. Both common and lethal, its estimated worldwide incidence is 31.5 million sepsis cases. Of these, 19.4 million are severe, causing 5.3 million deaths annually [2]. In the UK, it is estimated that 7.7 % of deaths are associated with sepsis [3]. Reported mortality rates for all forms of sepsis vary by country and case mix, ranging from 15 to 80 % [4–6]. Hospitalisation rates for sepsis in the developed world are increasing, and substantially outweigh those for stroke and myocardial infarction combined [7]. Sepsis also remains the leading cause of maternal mortality in the UK [8]. In addition to its impact on mortality, the physical and psychosocial sequelae of sepsis have long-term consequences for survivors’ quality of life [9].

Mortality from sepsis can be reduced by early and reliable recognition, permitting effective management [10]. Best practice includes the timely administration of broad-spectrum antimicrobials within 1 h of presentation [11–13], based on evidence of a 7.6 % decrease in survival rate with every hour’s delay in administering antibiotics [11]. The impact of appropriate empiric antibiotic treatment on mortality is such that the number needed to treat to save one life is just 10 patients [14]. In the UK, improving the recognition and response to sepsis has become a national priority [15], supported by a number of initiatives. They include the National Confidential Enquiry into Patient Outcome and Death (NCEPOD) recommendations [16], the newly developed National Institute for Health and Care Excellence (NICE) guideline for sepsis [17], and the introduction of financial incentives for delivery of antibiotics within an hour via Commissioning for Quality and Innovation (CQUIN) targets [18].

Globally, concerted efforts to reduce the harm from sepsis have accelerated in the last 15 years. In 2002, the international Surviving Sepsis Campaign was launched, focusing initially on agreeing and disseminating evidence-based recommendations for treating and managing sepsis. In order to simplify implementation, the Campaign identified two ‘bundles’ of interventions for treatment and management, comprising evidence-based elements, which could readily be measured, audited, and incorporated into a checklist. The Campaign promoted a bundle of evidence-based interventions to be completed within 3 h of presentation (measure lactate; obtain blood cultures; administer antibiotics and crystalloid) and a bundle of interventions to be completed within 6 h if appropriate (apply vasopressors, re-assess volume status, re-measure lactate) [19, 20]. By distilling the recommendations into such bundles, it was anticipated that implementation would be more ‘feasible with proper motivation and organization.’ Timely completion of these bundles has been found to be associated with reduced mortality [21, 22].

Despite their vaunted simplicity, however, compliance globally with both bundles is weak, [23, 24] with an international audit in 2015 finding compliance worldwide with 3-h and 6-h bundles of just 19 and 36 %, respectively, though this varied significantly by country and continent [24]. Accordingly, recognition has increased that the ‘bundling’ of recommendations does not in itself guarantee implementation, and researchers have called for greater measures to support improvement, such as education programmes and dedicated response teams [23]. A recent systematic review and meta-analysis of such performance improvement programmes found that they were associated with increased bundle compliance, but with significant variations in effectiveness reflecting the diversity of approaches taken [22]. Distinguishing between ‘educational’ and ‘process change’ approaches, it found that programmes that incorporated both approaches were more effective in increasing compliance than programmes that included only one or the other [22].

The ‘Sepsis Six’ clinical care bundle, promoted by the Survive Sepsis organisation in the UK, builds on the bundles developed by the international Surviving Sepsis Campaign [25], focusing solely on six key tasks required to treat a patient with sepsis, including prompt delivery of first antibiotic dose along with two other core therapeutic interventions (administering oxygen therapy and intravenous fluids) and three diagnostic and monitoring steps (blood cultures and measuring urine output and lactate) (Table 1) [26].

**Table 1:** The Sepsis Six

1. Deliver O_2_ (oxygen) to target 94–98 % SpO_2_ (oxygen saturation) or 88–92 % in COPD (chronic obstructive pulmonary disease)	
2. Take blood cultures	
3. Give IV (intravenous) antibiotics	
4. Start IV fluid resuscitation	
5. Measure serum lactate	
6. Monitor urine output	

Delivery of the Sepsis Six bundle within an hour of recognition of sepsis is recommended as an approach to improve the effective management of septic patients. Consistent with the international picture, however, compliance with the Sepsis Six remains poor, and interventions to improve reliability of completion have shown only modest success. One audit of a campaign to promote improved response to sepsis found that all six steps were completed in only 23 % of patients with sepsis, although 89 % received the treatment elements of the bundle within 4 h [27].

In many ways, the experience of the Sepsis Six is no surprise: one of the most consistent findings in health care is the difficulty of introducing evidence and innovations into routine practice [28]. Programmes to implement interventions often fail to achieve expected improvements. One problem is often a lack of understanding of how interventions actually work and what is required to make them work [29, 30]. Even when an intervention is seemingly simple, like the Sepsis Six, the underlying changes that are required to embed it into practice, including widespread behaviour change, may be complex and require careful choice of implementation strategy [31, 32].

This indicates a need for a more nuanced understanding of the realities of implementing the Sepsis Six at the front line of care to inform implementation approaches. Yet to date, research of this nature has been notable by its absence: the well-documented inconsistencies in compliance and limitations of efforts to improve completion have not been accompanied by examination of what makes timely sepsis treatment so challenging, and the extent to which improvement programmes address these barriers. Based on an ethnographic study of a national collaborative programme on sepsis in Scotland, our study represents, to our knowledge, the first attempt to identify and characterise implementation strategies used to promote timely completion of the Sepsis Six and explore unanticipated challenges arising in the day-to-day practice of implementation.

## Methods

We report a grounded theory study, using focused ethnography, of efforts to manage patients with sepsis in hospitals in Scotland. The study was conducted as part of an evaluation of the Scottish Patient Safety Programme (SPSP) sepsis VTE (venous thromboembolism) collaborative. This collaborative programme incorporated a workstream that aimed to improve the recognition, timely resuscitation, and outcomes of patients with sepsis, through promoting the use of Early Warning Scores (EWS) [33] incorporating clinical sepsis criteria, and the application of the Sepsis Six clinical care bundle within an hour of presentation. Participating sites were trained in established quality improvement methods (e.g. Plan Do Study Act (PDSA) cycles and process mapping). Implementation approaches were not mandated; the ethos of the collaborative was that sites should generate their own local approaches to implementation and sharing learning with other sites. The collaborative provided central support to the teams through learning sessions, site visits and webinars and collected and fed back process and outcome data. All 15 territorial health boards caring for inpatients across Scotland participated in the collaborative; local teams were tasked with implementing improvement initially in one or two pilot wards or units before spreading interventions more widely.

We conducted observations of practice, and staff interviews, in a sample of six pilot sites selected to include diversity in terms of geographical location and size (number of acute beds). Fieldwork was conducted between March 2013 and May 2014. A researcher (BOD) visited each site for a period of around 5 days to conduct observations of front-line practice. Observation periods covered daytime and late evenings. The researcher shadowed staff involved in the care of acute medical patients, including doctors, ward-based nurses, and local response teams. She conducted observations in acute medical receiving units, emergency departments, and medical and surgical wards. The researcher observed ward rounds, handovers, safety briefs, and day-to-day patient care, with a particular focus on the management of patients with sepsis. She also observed relevant meetings and education sessions on sepsis. A second round of observational visits, each lasting 2 to 3 days, was conducted in three of the sites at a period of 9 to 12 months following the original visits to explore sustainability of improvement. Posters and information sheets were used to inform staff and patients about the study. Data were recorded in the form of written and audio-recorded fieldnotes, supplemented by recordings of research team debriefing sessions. We reviewed process and outcome data related to sepsis produced by each site as part of the collaborative work.

Semi-structured interviews were conducted with a purposive sample of staff in each site, including members of the local collaborative team, and frontline staff in a range of roles. Interviews were conducted face-to-face, with a small number conducted by telephone. Informed consent was obtained prior to interview. Interviews explored staff experience of participating in the collaborative programme and their experiences of implementing improvement locally. A topic guide was used flexibly, tailored to each individual staff member’s experiences (sample topic guide is shown in Additional file 1). Interviews were digitally recorded, transcribed verbatim, and anonymised.

Data analysis drew on the constant comparative approach [34]. A sample of fieldwork and interview transcripts were initially open coded; codes were then grouped together iteratively and revised to develop a full thematic coding frame, which was applied to subsequent transcripts. NVivo 10 software was used to manage the coding and analysis process. Data summaries of themes were prepared, and text segments within and across themes compared, and summaries interrogated, to generate a final narrative. We reflected on and discussed each site’s data on their implementation rates (including % Sepsis Six within 1 h) in relation to our qualitative analysis, but due to the variable quality and completeness of this data, we did not undertake a comparative quantitative assessment of the performance of each unit.

## Results

We conducted around 300 h of observations across a range of settings in the six pilot sites. Interviews were conducted with 43 members of staff in different roles: consultants, senior nurses, staff nurses, managers, trainee doctors, and a pharmacist. The researcher was able to observe real-time responses to around seven patients with suspected/confirmed sepsis in each site, as well as discussing with staff other cases identified from local documentation.

### Implementation strategies

Sites engaged to varying degrees with Quality Improvement methodology. PDSA cycles were commonly used, particularly to develop and implement new documentation, but there was little engagement in more complex diagnostic work, such as barriers/facilitators assessment, to underpin the selection of implementation strategies, and little evidence of explicit use of theory to design implementation approaches. The local work to implement the Sepsis Six primarily focused on educating staff about sepsis and the Sepsis Six, promoting staff engagement in improving the detection and management of sepsis, and maintaining a high profile for sepsis in day-to-day practice, for example, through daily reminders in ward rounds and in safety briefs.

We are doing a rolling education programme for the nurses and that’s three hours […] talking about Sepsis Six, […] and a case study on patients. (Interview, consultant, Site 1)

Dissemination from the charge nurses just to keep stating this is what we’ve got to do, just make sure it gets done. (Interview, senior nurse, Site 3)

A common intervention was to use strategies based on prompts and reminders, such as introducing posters and Sepsis Six stickers for patient notes, to act as cues to staff. There was also a strong focus on motivating staff to achieve targets of completing the Sepsis Six within an hour, and rewarding them when they did so: the use of timers or competitions for time to first antibiotic, and feedback of performance data and celebration of improvement, were commonly used strategies to motivate staff. There was some evidence that staff appreciated these strategies and responded well to positive feedback:

It’s audited […] on a weekly or monthly basis by one of the registrars. So they come back to us and, with a chart to show us how well we’re doing. […] It makes you feel quite good actually when you know you’re up there [i.e. have high compliance with the Sepsis Six]. (Interview, senior nurse, Site 1)

Completing the Sepsis Six also required coordination of equipment and resources, and sites commonly tried to tackle the issue of making equipment easy to access once the Sepsis Six was initiated, by setting up a sepsis trolley or drawer which would contain all the necessary equipment and documents for staff who were delivering the Sepsis Six.

We’ve got a sepsis drawer at each bed space in triage which has got your fluids, your giving sets, your packs, all your blood forms, your blood tubes. Your blood form has been pre-printed so that we have all our requests on it. […] We’ve got all the antibiotics in a box in the cupboard in triage so that they’re easily accessible. (Interview, senior nurse, Site 2)

These strategies were successful at raising staff awareness of sepsis and engagement with implementing the Sepsis Six, reflected in perceptions of a shift in culture and attitudes towards the management of sepsis. According to their own data collection exercises, all sites saw improvements in their rates of completion of the Sepsis Six associated with the introduction of interventions.

A student nurse […] said that when there was a case of sepsis everyone is like ‘sepsis, sepsis, sepsis!’ (Fieldnotes, Site 1)

[Consultant] said […] in April 2012 we were at 25 % [of patients receiving Sepsis Six within an hour] and now it is 75 % and that is great. (Fieldnotes, Site 4)

Our study identified that while sites used a combination of interventions to improve reliability of delivery of the Sepsis Six, intervention choice heavily favoured interventions that targeted individual behaviour change—increasing knowledge, raising awareness, motivating staff, and restructuring the environment to facilitate the desired behaviour (i.e. through ensuring equipment was at hand in a pack or trolley). This was predicated on the belief that overcoming these cognitive and motivational barriers was the principal task—and that once this achieved, the implementation of the individual steps would follow relatively easily.

There is absolutely nothing complicated about managing a septic patient (Interview, Senior Charge Nurse, site 1)

Our emergent theory indicated, however, that despite the beguiling apparent simplicity of the individual steps in isolation, the process of completing the Sepsis Six within an hour was far from straightforward and could fail in unanticipated ways.

### Completing the Sepsis six: a series of six simple steps?

On superficial examination, it appears that the Sepsis Six involves six simple steps. In practice, it was evident that completion actually involved a complex trajectory [35], comprising multiple interdependent tasks that required prioritisation and scheduling between staff and across boundaries within organisations. A composite example of the processes involved is given in Table 2.

**Table 2:** Composite example of steps involved in Sepsis Six, observed in fieldwork

1. Administer high flow oxygen • Find a doctor, get oxygen prescribed • Assess for COPD • Gather together equipment • Wash hands • Explain the reason for the procedure & gain consent • Administer O_2_ and check for comfort • Document 2. Take blood cultures • Find a doctor/nurse/phlebotomist who can take the bloods • Gather the equipment • Explain the procedure to the patient & gain consent • Wash hands/gloves • Carry out the procedure • Leave the patient comfortable • Send off the samples • Document 3. Give IV antibiotics • Find a doctor—get the antibiotics prescribed and check for any allergies • Find a nurse with IV certificate, wash hands and prepare the medication • Ensure the medication is checked by two nurses • Gloves • Take the medication to the patient with the drug record • Explain the procedure and gain consent • Check the patient’s identity at point of administration • Administer the medication and observe for any adverse effects • Document	4. Start IV fluid resuscitation • Find a doctor/nurse who can cannulate, get IV access • Gather equipment • Wash hands • Check the prescription and the fluids and set up the infusion • Explain the procedure to the patient and gain consent • Gloves • Connect and commence the fluids • Ensure patient comfort • Remember the PVC care bundle 5. Measure serum lactate • Find a doctor or a nurse who can take a venous lactate • Gather equipment • Wash hands/ Gloves • Explain the procedure and gain consent • Obtain the sample • Ensure patient comfort • Find a lactate machine • Get the result, inform the doctor and document 6. Monitor urine output • Explain why and ask the patient to pass urine • Wash hands • Assist them to the toilet/commode • Help the patient back to bed, remember to save the sample! • Wash hands/gloves • Go back and get sample, measure it, test it and send off sample • Find a fluid balance chart and record

Carrying out the tasks required to complete the Sepsis Six within the hour involved the coordination of multidisciplinary inputs. A common model for undertaking the Sepsis Six was for this to be driven by ward nurses, with support from (mainly trainee) doctors. Depending on the characteristics of individual patients, completing some elements of the Sepsis Six could require significant input from several staff.

One of the staff told me that the main challenges to the implementation of the Sepsis Six were difficulty obtaining venous access, or if the patient can't be stabilised, or if they have a difficult patient. This [particular] patient was quite dehydrated so access was difficult to obtain. […] Obtaining access [took] a FY1 [foundation year 1 trainee doctor], a consultant, a staff nurse and a charge nurse. (Fieldnotes site 1)

Staff commonly had to attend to completing the Sepsis Six in the context of a busy workload and had to prioritise their time across a set of competing demands. In the process, staff had to interweave the multiple (and often urgent) tasks required across the care of the many patients on a ward [36], and often experienced interruptions in their workflow [37, 38], meaning that they could not always prioritise or devote their attention fully to the task of completing the Sepsis Six.

FY1 […] said sometimes when you start doing [Sepsis Six] and the ward’s very busy it’s very difficult to get someone to help you to get everything done within the hour. (debrief site 2)

My biggest challenge is when you get lots of ill patients in at the same time. And you don’t have enough staff, and something’s got to give, and I get really concerned that [it] is actually quite often the giving of the antibiotics, which is the most important part, and quite often that’s the bit that’s missed. (Interview, Senior nurse, site 2)

Prioritisation of sepsis cases was a particular problem in emergency departments, where staff were engaged in a constant process of triage under significant time pressure. Patients with conditions that presented a visible and immediate threat to life were prioritised, meaning that in some cases, treating patients with suspected sepsis, who often did not present as seriously unwell, was delayed in favour of responding to patients with more visibly urgent demands. In addition, the prerogative to treat patients with sepsis sometimes came into conflict with other pressures around disposition of patients. In particular, the risk of negative consequences for breaching the 4-h target for emergency department wait in place across Scotland loomed larger than the consequences of delayed antibiotic administration, even though these consequences included an increased risk of mortality [39].

We have feedback from the nurses saying they just don’t have time in A&E [Accident & Emergency] to do [Sepsis Six]. […] People have got other clinical priorities and they think ‘well they’re not that sick, so I’m just gonna help somebody else. […] I’ll give them antibiotics in a minute. I’ll give them antibiotics when I’ve got time.’ (Interview, consultant, site 1)

[The charge nurse] told me that they are under so much pressure from their 4-hour breach time […] that if the patient has five minutes until they breach and then they get asked to implement the Sepsis Six, they are then under pressure from senior management to move the patient on. (Fieldnotes, site 1)

Completing the Sepsis Six was complicated by the task interdependence inherent in the sequence of tasks required: sequential interdependence (where one task needs to be completed before another) and reciprocal interdependence (where individuals are dependent recursively on each other to perform tasks) [40]. The conditions for achieving these interdependencies were precarious, and staff were easily disrupted by interruptions, competing pressures, and the discontinuities created by transfer of patients. Nurses in most cases were reliant on doctors to prescribe antibiotics, and on other nurses to check and sign, before they could administer the antibiotics to the patient. Not all ward nurses had the skills, permission, or authority required to start fluids in patients, or to insert peripheral venous catheters: they had to call on other staff with the expertise or authority to complete these tasks, potentially introducing delays.

Nurses […] have to say ‘well a patient needs antibiotics, and […] can they get fluids put up?’ […] So they need the direction from the doctor [in order to start treatment]. (Debrief, site 6)

Not all our nursing colleagues are trained in PVC [peripheral venous catheter] insertion, so all PVC insertion is done by doctors. (Interview, consultant, site 4)

Coordination of activity around shared tasks involves communication and collaboration; failures in communication, particularly between doctors and nurses, were a significant source of weakness in the reliability of the process. Communication breakdowns sometimes resulted in significant delays in antibiotics being administered, as nurses were not always aware that doctors had prescribed them.

This is the bit that I find really frustrating […] the communication. […] [Doctors] seem to think that nurses can read through folders, and we’re trying to get them to understand that if you write up a stat [immediate] dose of antibiotics you must tell a nurse as soon as you do it, and it’s done. Because it’s maybe an hour later [the doctor will] think ‘have you given that antibiotic’, [But the nurses say] ‘Did you prescribe it? You never told me!’ (Interview, non-clinical lead Site 2)

The patient did end up getting a lactate done and the blood results came back with a raised white cell count and the doctor went and prescribed the antibiotics, but […] hadn't told any of the nursing staff (Debrief, site 6)

Additionally, completion of the Sepsis Six was commonly disrupted by operational failures— failures that stem from the ‘the work system […] or the actions of others that supply workers with necessary materials, information or services’ [41].

Then the charge nurse told me that if there's a delay in returning the blood results then again this would impact on Sepsis Six being implemented. (Debrief site 1)

A set of observations from an acute medical unit (Table 3) illustrates how, although staff were working on the Sepsis Six with a patient, competing tasks and priorities, distractions from other patients, and lack of synchronisation of inputs into interdependent tasks meant that the patient did not receive antibiotics until around three hours after they were initially prescribed. The extract highlights the multiple tasks required to complete the Sepsis Six (including the need to assess patients’ weight, and consider antibiotic choice for the presumed sepsis prior to prescribing) and the conflicting and competing demands on doctors’ and nurses’ time and attention as they try to complete these tasks.

**Table 3:** Delay in administering antibiotics

4.25 pm- [Following prompt initiation of the Sepsis Six] the consultant has prescribed one of the antibiotics.
4.45 pm Antibiotics are still not given. The nurse has moved on to another patient who has just been admitted.
5.05 pm- [FY1] asks how much the patient weighs. They can’t weigh him so he is going to estimate it. He is going to give the patient Gentamicin as well as the Amoxicillin. I watch as he reviews the consultant’s notes. Meanwhile the nurse is putting a bag of fluids up. I hear the FY1 ask the patient some questions about where he is [i.e. undertaking a dementia assessment]. The nurse then tells me she will give the antibiotics once the doctor tells her the dose of Gentamicin.
5.20 pm-The doctor prescribes the Gentamicin. The nurse gets caught up with relatives from another admission as the [new] patient has dementia and can’t tell her anything.
6 pm- The nurse is still with the relatives.
6.30 pm - Both the antibiotics are signed for by two nurses, but a nurse is still not available to administer them.
7.15 pm- Finally I watch as the nurse administers the patient’s antibiotic, almost 3 hours after they were first prescribed (Fieldnotes, Site 4)

### Addressing problems of coordination and operational failures

Despite the seeming intractability of these challenges, we identified some strategies that went beyond educating staff about sepsis and promoting engagement and went some way towards mitigating against coordination problems and addressing operational failures.

Systematic efforts in local sites to understand the complexity of task completion for the Sepsis Six, and of how task completion could be frustrated by problems in workflow and systems, proved extremely valuable in informing improvement. One site used process mapping to identify failures along the Sepsis Six pathway and was able to identify critical operational weaknesses and failures, some of which were relatively simple to resolve once the nature of the underlying problems had been identified.

In some places we haven’t had lactates within the hour so we’ve had to purchase modules on blood gas analysers. […] So actually identifying through the process mapping that, you know, we couldn’t achieve the lactate. So we’ve had to fix that. (Interview, non-clinical lead, Site 6)

Changes to the way responsibilities for initiating and completing the Sepsis Six were allocated could improve implementation by reducing the need for coordination and task-switching. The use of a response team to manage patients with suspected sepsis was a common model. These teams were usually led by an advanced nurse practitioner and were on standby to respond to deteriorating patients; this team would take over the responsibility for diagnosing sepsis and initiating the Sepsis Six. The team could be called by a ward-based nurse or doctor when a patient was identified as deteriorating. Additionally, in one site, members of the response team described ‘trawling the wards’ to identify deteriorating patients that they had not been alerted to. By concentrating the skills in one place and placing responsibility in a single team with clear responsibilities and without the distractions faced by other staff on wards and in emergency departments, this approach had promise in overcoming some of the operational challenges that faced others in delivering the Sepsis Six.

[Response team is] independently diagnosing, treating, prescribing [for] sepsis, and we actually don’t involve doctors in that process until we have finished and the patient has had antibiotics and fluids, because they were slowing us down. […] Unless we felt the patient needed to be escalated up to a critical care unit, we wouldn’t actually involve a medic in that process at all. So we have gone from zero to hero in two years. (Interview, ANP, Site 3)

But, though response teams were seen as effective in expediting the Sepsis Six, establishing a response team required a significant investment of resources. A further disadvantage of this approach was that ward staff missed out on learning opportunities which could help them develop the necessary skills to manage a patient with sepsis.

In the big wards, the big medical wards that are really busy, they don’t have the skills or expertise, and they say, you know, ‘somebody will come and do it’. (Debrief, Site 6)

An alternative approach involved training ward nurses in the techniques critical for completing the Sepsis Six and empowering them to start treatment without the need to call for a doctor (for example, through the use of Patient Group Directions (PGDs) [42] to enable nurses to administer fluids to patients in certain groups without referring to a doctor for an individual prescription). This reduced task interdependence and the need for coordination. While this approach did not overcome the problem of task and time prioritisation for ward-based nurses, allocating the responsibility to the nursing team created a situation in which task interdependency was confined to a group that had a set of informal obligations to one another. This facilitated the development of a more collaborative and supportive approach, based on norms of reciprocity [43].

Nurses historically could never administer fluids on their own, that always had to be prescribed.[…] [Now] the nurses have the skills behind them of basic assessment and they know when it’s appropriate when to hang a bag of fluids without having a doctor’s prescription. […] All the nurses on the unit here now can take both arterial and venous blood gas samples. And antibiotics as well, again we have two nurse prescribers within the unit who can prescribe the antibiotics, so that’s reduced the delay of getting a doctor to prescribe. (Interview, senior nurse, Site 1)

Everybody mucks in, the minute you identify someone as being septic, everyone is hands on. (Interview, senior nurse Site 1)

## Discussion

Implementation strategies to promote reliable use of the Sepsis Six across the units in the hospitals we studied primarily focused on education, engaging and motivating staff, and providing prompts for behaviour, along with efforts to restructure the environment to ensure that equipment required to complete the Sepsis Six was readily available. Although these strategies were well received and appeared successful at raising staff awareness and engagement, particularly on acute medical wards, our study identified that improving reliability in completion of the Sepsis Six within an hour was not straightforward. Achieving reliability of processes in high-risk organisations is challenging because it relies on collaboration between multiple groups of professionals, requiring communication and constant adjustments [40]. The process of completing the Sepsis Six reflected the challenges inherent in any effort to improve reliability in a complex system. Our emergent theory indicated that completion of the Sepsis Six invoked interdependencies between staff and required coordination and cooperation: because of the need for input from staff with different types of expertise and authority to complete sequential tasks, and the need to maintain a thread of continuity and purpose in completing the whole trajectory. A lack of synchronisation could arise when staff had to resolve competing demands and manage interruptions to their time. Breakdowns in communication disrupted or delayed completion of tasks. Our study indicates that, rather than being a simple technical intervention, the Sepsis Six bundle involves a set of complex processes. The problem of improving reliability of response to septic patients can be seen as a complex endeavour that requires attention to problems of coordinating tasks, workflow, accountability, and expertise, in the context of a busy and complex environment.

Approaches to implementation of interventions tend to be pragmatic rather than theory or evidence based, and difficulties in achieving improvement are often attributable to failures to use informal and formal theory to inform the design of improvement approaches [44]. A key argument relates to the need to have a clear understanding of the underlying problems to be addressed in achieving change, their root causes, and the mechanisms through which the chosen interventions are likely to address these problems— to maximise the likelihood that the logic behind the selection of implementation approaches is sound [45]. Implementation approaches to Sepsis Six in this study tended to focus on individual behaviour and use behaviour change approaches focusing on motivating, equipping, and empowering staff to adhere to best practice (e.g. awareness raising, education, modelling, persuasion, reminders and prompts, and feedback). This finding is consistent with findings from other work studying the implementation of the Sepsis Six [32]. To this extent, the approach to implementation taken in the case-study sites we examined were well thought through and certainly were in line with psychological theories of behaviour change in implementation [31, 46]. However, our study demonstrates that, despite their success in improving the profile of sepsis, informing staff of the need for swift action, and motivating at least some staff to invest considerable time and effort in improving response, in other ways, the approach fell short—there were still routine failures in the completion of the Sepsis Six within an hour. This was because the challenges of prompt and reliable administration of the Sepsis Six did not arise solely from knowledge deficiencies, cognitive limitations, or lack of motivation of staff, which would require a focus on individuals as the locus of change. Rather, they also related to the socio-technical complexity of completing interdependent tasks requiring multiple individuals in different professional groups in a frenetic environment characterised by competing priorities. Despite evidence of widespread engagement and implementation of interventions targeted at behaviour change across the sites, problems remained in achieving reliability at completing the Sepsis Six within an hour; these problems commonly arose from challenges of coordination and workflow, but these issues were generally not recognised as targets for intervention in the majority of sites. Sites on the whole lacked insight into these problems, and there were few examples of strategies specifically targeting these broader issues relating to coordination of workflow among multiprofessional teams and weaknesses in systems and processes.

Developing theory-based approaches to improvement involve ‘a strategy of moving back and forth from the world of theory to the world of action’ [47] and a strength of this study lies in the use of ethnographic methods to gain a deep understanding of the realities of implementing the Sepsis Six in everyday practice. Combining interviews with observations allowed us to explore the views, opinions, and experience of staff to help make sense of what was observed. Our data comes from a diverse sample of six hospitals in Scotland that varied in teams of size and locality.

Our study is limited by the methodological approach; ethnography is time and resource intensive, and we were only able to observe a selection of the sites that were participating in the collaborative. Our observations of the Sepsis Six were limited to those which happened to occur during our fieldwork visits. We were able to reflect on and discuss sites’ own data on their implementation rates, to inform our analysis, but the variable quality of this data meant we were limited in the extent to which we could draw conclusions about the overall performance of each site. Despite this, our qualitative approach provides a rich insight into the realities of efforts to improve practice at the front line of care and the unanticipated challenges that arise. We engaged in strategies to ensure the robustness of our data and analysis, including the use of a structured observation guide, and the collection and recording of detailed fieldnotes and documents. The use of interviews alongside observations acted to triangulate our fieldnotes. Credibility of data analysis was strengthened by a focus on reflexivity on the part of the researcher, the use of NVivo to support coding and analysis, and an iterative approach to analysis which cycled between intensive data coding, review and discussion between the research team, and writing of interpretative summaries. Themes were generated through close analysis and checking between CT (a social scientist) and BOD (a clinician), and emerging themes were checked for credibility by clinical members of the team. Our study is also limited in that it was conducted in one country within the UK; the impact of the collaborative, and the approaches to implementation, were to some extent shaped by the national context. One important feature of the Scottish context is the recent history of centrally led, and broadly successful, efforts to improve the quality and safety of healthcare on a national scale through the SPSP. The sepsis work reported in this paper did not start from a ‘blank page’ but built on earlier SPSP work on safety briefs, the SBAR (Situation Background Assessment Recommendation) communication tool [48], and EWS to improve recognition of deteriorating patients and multidisciplinary communication. Nonetheless, we suggest that our work has value in identifying and characterising the strategies that appear to be implicated in achieving improvement.

Our findings shed some light on the wider evidence base for interventions to implement sepsis bundles, which, as noted above, has to date been dominated by quantitative-associational studies. A recent systematic review of programmes to support implementation of the Surviving Sepsis Campaign’s bundles distinguished between ‘educational’ and ‘process change’ approaches and found that programmes that incorporated both yielded on average a higher increase in compliance than those that included only one or the other [22]. This finding echoes our own finding that the challenges associated with Sepsis Six completion reflect more than just a deficit of knowledge that educational interventions alone might address. However, the approaches classified by the review’s authors as constituting ‘process change’ were highly heterogeneous: they ranged from decision support tools, through changes in equipment availability, to introduction of dedicated teams or units. The authors did not disaggregate their analysis to examine the differential effect of these approaches. Our study suggests that challenges of coordination and interdependence—of the kind that might be addressed by alternative organisational approaches such as dedicated teams and individuals—were more problematic than challenges of clinical identification and decision-making. We suggest that future efforts at ‘process change’ focus on developing and evaluating interventions that address these problems of coordination in particular, as our study would suggest that this is where the most vexed challenges lie.

Our study demonstrates the importance of fully understanding the nature of interventions and the challenges in getting them to work in practice. Theory-based implementation is not just about amassing generic evidence about which implementation techniques are most effective [49], but also about understanding the nature of the problem, and using theory to drive selection of implementation strategies. This requires context-specific programme theories of change [44], as well as work to diagnose problems and select suitable solutions. In this study, we found that, in settings where efforts were made to diagnose and characterise the nature of the problem, seemingly intractable difficulties started to become possible to address—for example, through process mapping that identified the key flaws in the system that were preventing timely lactate availability. Using systematic methods to characterise the complexity of apparently simple tasks may help shed light on where problems lie [50]. This is not to say that all such problems will be readily solvable, but it is to argue that careful examination of the particular challenges faced in particular settings will at least mean that the right kind of solution will be revealed and avoid the waste of time and resources in a futile attempt to apply the wrong solution to a misconstrued problem. Selection of implementation strategies should be based on a full understanding of the roots of problems; theoretical frameworks such as the Consolidated Framework for Implementation Research [51] provide a potential basis for tailoring the selection of theory-based interventions and implementation strategies to match the problems that need to be solved [52].

While problems in achieving reliable implementation of the Sepsis Six may be complex to resolve, our study identified areas of good practice. We observed examples in which teams had implemented changes that helped overcome some of the vulnerabilities in the trajectory, such as the use of PGDs to reduce task interdependence. The notion that some of the problems faced by a community of practitioners trying to reduce mortality from sepsis could be addressed using knowledge and resources within a community points to the potential value of a positive deviance approach to improving practice. Positive deviance approaches aim to identify the characteristics of practice shown by high performers and to disseminate this knowledge within a community; the success of this approach in achieving reductions in door-to-balloon time in acute myocardial infarction is well documented [53], highlighting its applicability to other complex care trajectories, such as the Sepsis Six.

## Conclusions

This study highlights that, rather than being a simple technical intervention, the Sepsis Six involves a series of complex processes. Implementation efforts that focus on individual behaviour change are important in improving uptake of the Sepsis Six, but understanding the completion of the Sepsis Six as a complex trajectory of work highlights that improving reliability also requires attention to coordination of workflow and systems and to the mundane problems of interruptions and operational failures that get in the way of task completion. Theory-based approaches to implementation of improvement in responding to patients with sepsis are needed that go beyond a focus on individual behaviour change and that are based on thorough assessment of barriers and challenges to implementation.

## Additional file

Additional file 1: Topic guide for interviews with frontline staff. (DOCX 157 kb)

## Abbreviations

A&E: Accident & Emergency
COPD: Chronic obstructive pulmonary disease
CQUIN: Commissioning for Quality and Innovation
EWS: Early warning scores
FY1: Foundation year 1 trainee doctor
IV: Intravenous
NCEPOD: National Confidential Enquiry into Patient Outcome and Death
NICE: National Institute for Health and Care Excellence
O_2_: Oxygen
PDSA: Plan Do Study Act
PGD: Patient Group Direction
PVC: Peripheral venous catheter
SBAR: Situation Background Assessment Recommendation
SpO2: Arterial oxygen saturation
SPSP: Scottish Patient Safety Programme
VTE: Venous thromboembolism
