# Supplementary material for: A complex endeavour: an ethnographic study of the implementation of the Sepsis Six clinical care bundle
Source: Implement Sci. 2016 Nov 16;11:149. doi: 10.1186/s13012-016-0518-z (PMC5112724; doi:10.1186/s13012-016-0518-z)
Supplement: Additional file 1: — Topic guide for interviews with frontline staff. (DOCX 157 kb) [file 13012_2016_518_MOESM1_ESM.docx]

## Appendix 1. Topic guide for interviews with frontline staff


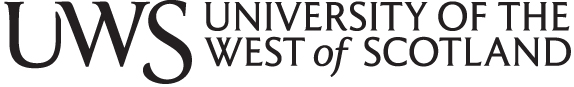

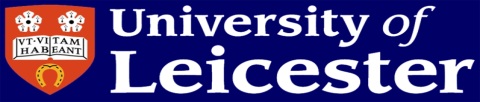


**Topic Guide**

**All participants**

- What is your current job title? How long have you worked here?
- Can you tell me how your clinical area came to be involved in the Sepsis/ VTE collaborative?

**SHARP END- For staff who have no experience of the collaborative programme but are involved in the delivery:**

- Do you think sepsis / VTE are a major problem in your area? Do you get any feedback on rates of sepsis and VTE?
- What do you know about the sepsis / VTE collaborative? What has this involved for you locally?
- What does the Sepsis Six mean to you?
- What does VTE risk assessment mean to you?
- How were these tools introduced to your clinical area? How do you use them?
- How do you feel about using these tools? Why? Do you think they are making a difference? Why?
- What else are you doing locally to tackle sepsis / VTE? Why? How do you feel about this?
- What are the difficulties in tackling sepsis and VTE?
- Is there anything about this ward / hospital / board that make it easier or more difficult to improve practices relating to sepsis / VTE?

**All participants**

- How do the sepsis VTE collaborative/ Sepsis Six/ VTE risk assessment impact on your daily work?
- Can you tell if you believe that your personal circumstances have any impact on your ability to participate in new initiatives/ programmes within your work place?
- What is it about your hospital that makes the collaborative work / not work?

**Effectiveness & Sustainability**

- What does effectiveness mean in clinical practice?
- What would make the sepsis VTE collaborative effective?
- Do you think that the new practices that are being implemented, due to the Sepsis VTE collaborative, will be sustained? Why? How long for?
- When changes come along, like the introduction of new tools/ practices to reduce sepsis, what do you think is needed for people to really get engaged and for the changes to ‘stick’?
- Do you consider that there is any connection between effectiveness and sustainability? Tell me what you think?
